# The role of the kynurenine pathway in cardiovascular disease

**DOI:** 10.3389/fcvm.2024.1406856

**Published:** 2024-05-31

**Authors:** Yuehang Yang, Xing Liu, Xinyi Liu, Chiyang Xie, Jiawei Shi

**Affiliations:** Department of Cardiovascular Surgery, Union Hospital, Tongji Medical College, Huazhong University of Science and Technology, Wuhan, China

**Keywords:** kynurenine pathway, cardiovascular diseases, coronary atherosclerosis, arterial calcification, myocardial diseases

## Abstract

The kynurenine pathway (KP) serves as the primary route for tryptophan metabolism in most mammalian organisms, with its downstream metabolites actively involved in various physiological and pathological processes. Indoleamine 2,3-dioxygenase (IDO) and tryptophan 2,3-dioxygenase (TDO) serve as the initial and pivotal enzymes of the KP, with IDO playing important and intricate roles in cardiovascular diseases. Multiple metabolites of KP have been observed to exhibit elevated concentrations in plasma across various cardiovascular diseases, such as atherosclerosis, hypertension, and acute myocardial infarction. Multiple studies have indicated that kynurenine (KYN) may serve as a potential biomarker for several adverse cardiovascular events. Furthermore, Kynurenine and its downstream metabolites have complex roles in inflammation, exhibiting both inhibitory and stimulatory effects on inflammatory responses under different conditions. In atherosclerosis, upregulation of IDO stimulates KYN production, mediating aromatic hydrocarbon receptor (AhR)-induced exacerbation of vascular inflammation and promotion of foam cell formation. Conversely, in arterial calcification, this mediation alleviates osteogenic differentiation of vascular smooth muscle cells. Additionally, in cardiac remodeling, KYN-mediated AhR activation exacerbates pathological left ventricular hypertrophy and fibrosis. Interventions targeting components of the KP, such as IDO inhibitors, 3-hydroxyanthranilic acid, and anthranilic acid, demonstrate cardiovascular protective effects. This review outlines the mechanistic roles of KP in coronary atherosclerosis, arterial calcification, and myocardial diseases, highlighting the potential diagnostic, prognostic, and therapeutic value of KP in cardiovascular diseases, thus providing novel insights for the development and application of related drugs in future research.

## 1 Introduction

**Figure 1:**
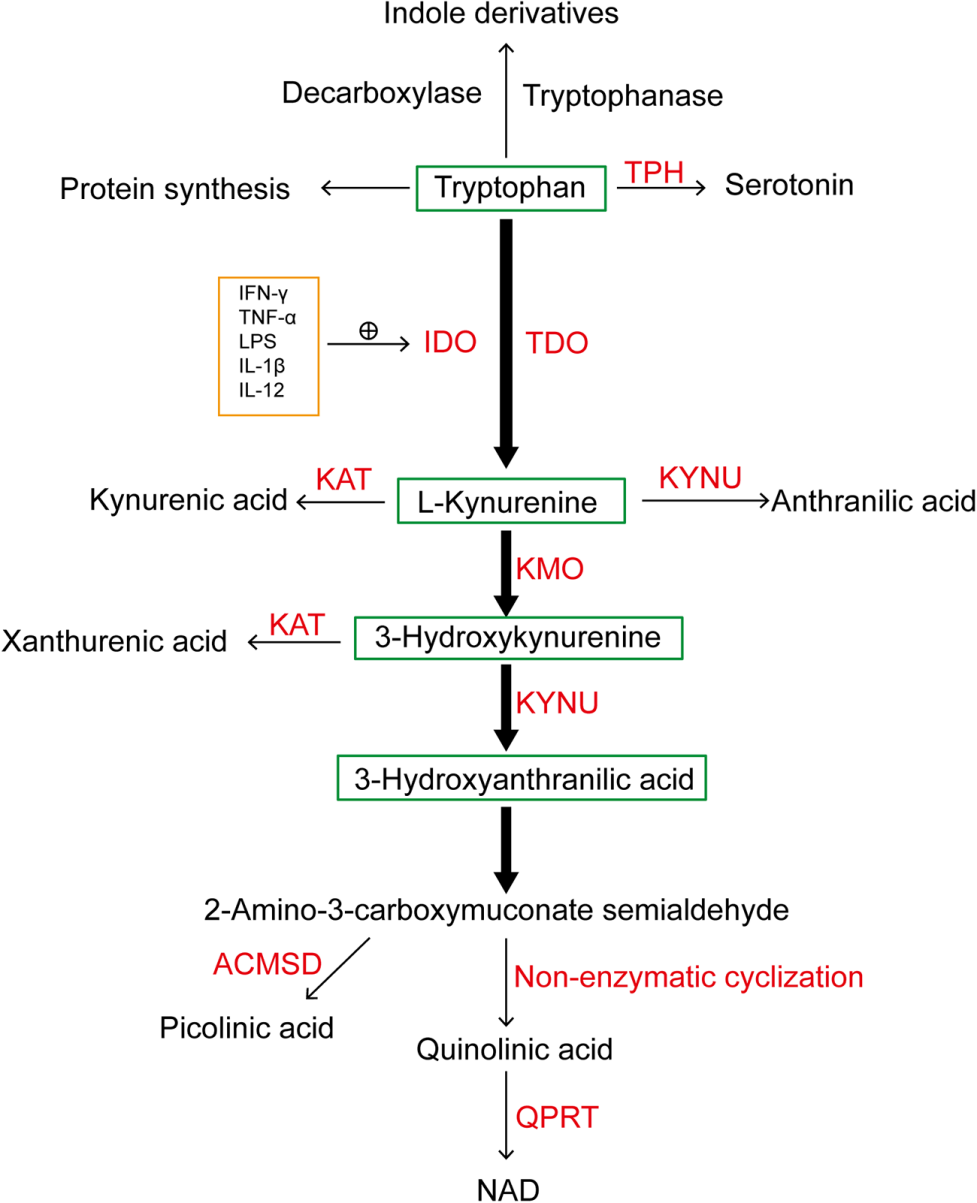
A simplified diagram illustrating tryptophan metabolism, with the majority of tryptophan metabolized through the kynurenine pathway under the catalysis of IDO and TDO. TPH, tryptophan hydroxylase; IDO, indoleamine-2,3-dioxygenase; TDO, tryptophan-2,3-dioxygenase; KAT, kynurenine aminotransferase; KYNU, kynureninase; KMO, kynurenine 3-monooxygenase; NAD^+^, nicotinamide adenine dinucleotide; QPRT, quinolinate phosphoribosyl transferase; ACMSD, aminocarboxymuconate semialdehyde decarboxylase; IFN-γ, interferon-γ; TNF-α, tumor necrosis factor-α; LPS, lipopolysaccharides; IL-1β, interleukin-1β; IL-12, interleukin-12.

Tryptophan (TRP), an essential amino acid, undergoes degradation in the body, generating a diverse range of bioactive substances. In mammals, over 95% of tryptophan degradation primarily occurs through the kynurenine pathway (KP), while the remaining 5% converts to serotonin via Tryptophan hydroxylase. The initial catalytic step involves indoleamine-2,3-dioxygenase (IDO) or Tryptophan 2,3-dioxygenase (TDO), predominantly yielding N-formyl-L-kynurenine. Subsequently, this compound undergoes further conversion into kynurenine (KYN). Normally, the basal degradation of the KP is primarily mediated by TDO in the liver, which is a tetrameric heme-based enzyme predominantly expressed in hepatic tissue, modulates serum TRP levels. Its expression can be induced by tryptophan itself, glucocorticoids, and estrogen (1). IDO manifests in two isoforms, IDO1 and IDO2. Despite both being heme-based enzyme, they regulate the extrahepatic branch of this pathway. Although the basal expression level of IDO1 within the human body is almost negligible, its presence is widespread. The activity of IDO1 can be further modulated through the activation of pro-inflammatory factors, including molecules such as interferon-*γ* (IFN-*γ*), tumor necrosis factor-α, lipopolysaccharide, interleukin-1β (IL-1β), IL-12, among others. This modulation contributes to the promotion of tryptophan metabolism in pathological states (2, 3). The breakdown of KYN initiates three distinct pathways. Firstly, KYN undergoes transformation into kynurenic acid facilitated by kynurenine aminotransferase. Secondly, KYN is converted into anthranilic acid by kynureninase. Thirdly, KYN degradation leads to the formation of 3-hydroxykynurenine through the action of kynurenine 3-monooxygenase (KMO). Subsequently, 3-hydroxykynurenine can either be further decomposed into xanthurenic acid by kynurenine aminotransferase or degraded by kynureninase to form 3-hydroxyanthranilic acid (3-HAA). The subsequent conversion of 3-HAA to 2-amino-3-carboxymuconate semialdehyde precedes two pathways: conversion to picolinic acid facilitated by aminocarboxymuconate semialdehyde decarboxylase and non-enzymatic cyclization to quinolinic acid (QA). Ultimately, the transformation of QA into nicotinamide adenine dinucleotide (NAD^+^) is catalyzed by quinolinic acid phosphoribosyl transferase (4–6) (Figure 1).

The mechanistic involvement of the KP in neurological disorders and cancer has been widely explored. KYN and its downstream metabolites play pivotal roles in modulating immune function and inflammation (7). Recent studies have highlighted the involvement of KP disorders in the development of cardiovascular diseases. Some researchers have observed the Kyn/Trp ratios (KTR) as an indicator, serving as a surrogate for the activity of the IDO1 enzyme and measuring KP activity. Elevated levels of KYN and 3-hydroxykynurenine have been detected, showing a positive correlation with inflammatory markers. Moreover, in patients with end-stage renal disease, the serum exhibited significantly increased KTR compared to that of normal subjects (8). The available findings indicate that elevated KTR is observed across various conditions, encompassing aging, adverse cardiovascular events, obesity, and type 2 diabetes mellitus (9). Furthermore, downstream metabolites of the KP have been identified as biomarkers for two significant diseases: coronary atherosclerosis and diabetes mellitus (10). The primary objective of this manuscript is to elucidate the role of the KP and its downstream metabolites in cardiovascular diseases, while delving into the molecular mechanisms underlying the interplay between KP and inflammation, atherosclerosis, arterial calcification, and myocardial diseases. Subsequently, it introduces the potential diagnostic and prognostic value of KP in cardiovascular diseases. Finally, the pharmacological mechanisms of KP in cardiovascular diseases are analyzed, aiming to furnish a theoretical framework for the development of pertinent therapeutics.

## 2 The kynurenine pathway

### 2.1 Kynurenine and kynurenic acid

KYN is extensively distributed in both blood and tissues throughout the human body, exhibiting varying levels among different organs. It can be produced internally by the human liver and obtained through various dietary sources. Among these, honey, lavender, sunflower, and mushrooms are rich sources of KYN (2). In addition, as per a study conducted by Anne-Lisa et al. In young female populations, serum concentrations of KYN and kynurenic acid are significantly lower in vegetarians compared to omnivores, with alanine aminotransferase levels also notably reduced (11). Furthermore, apart from its metabolic function, KYN serves as an agonist for the aromatic hydrocarbon receptor (AhR). The interaction between KYN and AhR may potentially reduce the risk of specific toxicants-induced diseases, thus serving as a protective mechanism for organisms. KYN enhances the differentiation of Treg cells and modulates immune responses by binding to AhR in the cytoplasm of T cells. Immunologically, AhR functions as a sensor for internal and external environmental cues, regulating inflammation accordingly. However, in the presence of a large number of exogenous ligands or ligands produced by tumor cells, AhR may impede the clearance of tumors by the immune system and contribute to the onset of autoimmune diseases (12, 13). In the context of aging and inflammation, chronic low-grade inflammation in the human body leads to aberrant activation of the KP. Inflammatory factors induce the upregulation of IDO1 expression, resulting in the accumulation of KYN and its downstream metabolites, thereby triggering certain inflammatory diseases and muscle wasting syndromes. Additionally, KYN can mediate the inhibition of osteogenic gene expression through AhR, thereby inducing osteoporosis in the elderly (14). KYN is acknowledged as a neuroprotective agent with the ability to traverse the blood-brain barrier and directly exert neuroprotective effects within the brain (15). kynurenic acid and QA traverse the blood-brain barrier at a relatively slower rate, primarily deriving from the metabolism of KYN within the brain. Surprisingly, research has reported that physical activity can expedite the conversion of KYN to kynurenic acid, thereby reducing KYN accumulation in various tissues. Physical exercise has been found to prevent and treat cancers, depression, and muscle wasting syndromes, while also enhancing energy metabolism, fatigue recovery, and antioxidant capacity (16–18).

Kynurenic acid can be produced in various types of cells and tissues, but it is primarily absorbed through the intestines into the bloodstream. It is predominantly distributed in physiological fluids such as urine, breast milk, bile, and serum in the human body (19). Similarly to KYN, kynurenic acid is also considered a neuroprotective agent within the nervous system. However, its limited ability to penetrate the blood-brain barrier impedes its direct development as a drug (20). Currently, research on the mechanisms of kynurenic acid primarily focuses on the nervous system, while its role in the periphery remains unclear. Kynurenic acid exerts anti-inflammatory effects by inhibiting various inflammatory pathways, including its activation of G protein-coupled receptor 35 (GPR35) to regulate cyclic adenosine monophosphate (cAMP) production and the activation of AhR to modulate diverse cytokines, thereby regulating immune responses (21). Studies have demonstrated kynurenic acid's capacity to diminish myocardial ischemia/reperfusion injury *in vivo* by binding to various receptors, manifesting myocardial ischemic protective effects. kynurenic acid interacts with GPR35 and subsequently translocates to the outer mitochondrial membrane, where it binds to the Adenosine 5′-triphosphate (ATP) synthase inhibitory factor subunit 1, reducing ATP loss during myocardial ischemia (22). Furthermore, this ischemic protective effect of kynurenic acid has also been observed in retinal ganglion cells (23).

### 2.2 QA、3-HAA and KMO

QA is primarily produced in microglial cells and macrophages in the brain, where it is mainly involved in neuroinflammatory lesions and oxidative stress (24). Under physiological conditions, QA typically remains below 100nM, promoting increased production of NAD within nerve cells. However, elevated levels of QA under pathological conditions can lead to neuronal dysfunction or death through various pathological mechanisms (25). QA, recognized as a neurotoxic substance, induces impaired glutamate reuptake in astrocytes by activating n-methyl-d-aspartate receptors, ultimately leading to neuroexcitotoxicity (21). Furthermore, QA contributes to oxidative stress by engaging in lipid peroxidation reactions and generating reactive oxygen molecules, posited as a component in the pathogenesis of Alzheimer's disease (26, 27). Within the immune system, QA demonstrates a pro-inflammatory effect, with experiments showcasing its abundant presence in immune cells (28). However, QA can also undergo metabolic breakdown to supplement intracellular NAD^+^, thereby combating infections and inflammation within the body. Additionally, quinolinic acid can alleviate skin inflammation by mediating an AhR-dependent mechanism (29).

Despite 3-HAA being acknowledged as a neurotoxic substance, it has shown the ability to efficiently cross the blood-brain barrier and trigger neuronal apoptosis (30). However, research has shown that 3-HAA can increase the percentage of Tregs by inhibiting Th1 and Th2 cells. It can also reduce inflammation induced by Th17 cells. Within blood vessels, 3-HAA can inhibit the uptake of low-density lipoprotein (Ox-LDL) in macrophages, thereby reducing local vascular inflammation and atherosclerosis. Furthermore, it can lower very low-density lipoprotein levels, exhibiting favorable lipid-lowering effects (31).

KMO, a mitochondrial enzyme belonging to the nicotinamide adenine dinucleotide phosphate hydrogen (NADPH)-dependent flavin monooxygenase family, operates with the flavin adenin dinucleotide cofactor and holds a central position within the KP metabolism (32). KMO is primarily present in peripheral tissues, macrophages, and monocytes, while in the nervous system, it is mainly concentrated in microglial cells (33). KMO catalyzes the transformation of KYN into neurotoxic compounds, notably 3-hydroxykynurenine and QA, known for their brain excitotoxicity (34). Elevated production of these toxic metabolites and free radicals in the bloodstream triggers neuronal apoptosis. The inhibition of KMO activity shifts KP metabolism towards the kynurenic acid branch, known for its neuroprotective effects (35). Research has revealed that inhibiting KMO activity leads to improved disease presentations in yeast, Drosophila, and mouse models of neurodegenerative diseases (36, 37). Experimental evidence suggests that the application of KMO inhibitors effectively reduces 3-hydroxykynurenine levels, alleviating inflammation in patients with severe pancreatitis. Additionally, it lowers the risk of mortality during multi-organ dysfunction syndrome (38) (Table 1).

**Table 1:** The respective roles of kynurenine and its downstream metabolites.

Compound	Blood-brain barrier crossing rate	Role in body tissues
KYN	fast	Direct neuroprotective effects (15). Detection of toxic substances in the body and reduction of the risk of diseases caused by them through activation of AhR (13). Activation of AhR exerts an immunosuppressive effect and promote the immune escape of tumor cells (13). Inhibition of osteoblast differentiation and promotion of osteoclast action (83). Enhance the differentiation of Treg cells, modulates immune response (12). Vasodilation (62).
QA	slow	Agonizes NMDA receptors leading to neurotoxicity (21). Promote oxidative stress (26). Alleviate skin inflammation through AhR (29).
KYNA	slow	Neuroprotective effect (37). Anti-inflammatory effect (21). Myocardial ischemic protective effect (22).
3-HAA	fast	Reduce local vascular inflammation and atherosclerosis (31, 97).
3-HK	fast	Neurotoxic, accumulates and damages nerve cells (23). Induced apoptosis of immune cells and temper inflammatory response (64).
KMO	slow	Catalyze neurotoxic compounds production (34). Exerts inflammatory modulation (38).

KYN, kynurenine; QA, quinolinic acid; NMDA, N-methyl-D-aspartate; AhR, aromatic hydrocarbon receptor; KYNA, kynurenic acid; 3-HAA, 3-hydroxyanthranilic acid; 3-HK, 3-hydroxykynurenine; KMO, kynurenine 3-monooxygenase.

### 2.3 Indoleamine-2,3-Dioxygenase 1

IDO1 is extensively distributed within the cytoplasm of various cells, its expression is notably higher in non-specific immune cells, including monocytes and macrophages (34). Its primary function involves promoting the production of KYN, thereby endogenously activating the AhR and regulating a series of immune responses (39). In the tumor microenvironment, KYN produced by IDO1 and TDO2 induces an immunosuppressive effect through AhR activation, influencing the tumor microenvironment and impairing the immune system's ability to recognize and eliminate tumor cells. This contributes to cancer cells' immune evasion (40). Studies have shown that IDO1 fosters tumor cell neovascularization by elevating IL-6 levels, counteracting the anti-cancer effects of the inflammatory cytokine IFN-*γ*, thereby promoting cancer progression. Similarly, in mice with IDO1 and IL-6 knockout, a decreased probability of tumor metastasis and improved survival rate were observed (41, 42). In response to these observed phenomena, researchers have developed a range of IDO1-selective inhibitors, including Epacadostat, Navoximod, BMS-986205, etc., currently undergoing clinical trials (43), these inhibitors hold promise for future tumor therapy. Furthermore, IDO1 serves the function of promoting vascular endothelial cell production, apoptosis, and reducing oxidative stress by synthesizing NAD^+^ (44). Studies indicate a close association between IDO1, KYN, and immunosuppression. In the context of a Th1-type immune response, activated T cells produce IFN-*γ*, in hhhducing increased IDO1 levels, consequently leading to elevated production of significant amounts of KYN and its downstream metabolites (45). These metabolites have been observed in mouse and human T cells, limiting the proliferation of specific Th1 cells while displaying less impact on other Th2 cells (46, 47).

### 2.4 Indole derivatives

An increasing body of evidence supports the close interplay between gut microbiota homeostasis and the cardiovascular system. Gut microbiota play a role in the pathogenesis of cardiovascular diseases by modulating immune responses, inflammation, and oxidative stress (48). In the gut, a small fraction of TRP can be metabolized by enzymes such as decarboxylase and tryptophanase to produce indole-3-acid-acetic, indole-3-aldehyde, indole-3-propionic acid, and indole-3-acetaldehyde, among other indole derivatives. These indole derivatives not only contribute to maintaining the integrity of the intestinal epithelial barrier but also possess antiplatelet and anticoagulant properties, thereby preventing thrombus formation and promoting myocardial repair. Recent studies have highlighted the potential role of indolebutyric acid, found in intestinal flora, in inhibiting the rise of KYN while simultaneously boosting the production of KYN downstream products such as xanthurenic acid and 3-HAA (49). Furthermore, these indole derivatives can serve as ligands for the AhR. AhR, as a crucial component of barrier immune responses in the gut, is closely associated with maintaining gut homeostasis (50). In the cardiovascular system, AhR promotes vascular and myocardial development, and is closely linked to myocardial hypertrophy, atherosclerosis, myocardial ischemia-reperfusion injury, and hypertension (51). Therefore, indole derivatives generated from tryptophan metabolism in the gut may mediate AhR's cardiovascular protective effects.

Intestinal dysbiosis can lead to activation of inflammasomes and disruption of the intestinal barrier. This disruption allows bacterial products or endotoxins to enter the bloodstream and trigger inflammation. Ultimately, the accumulation of various inflammatory stimuli contributes to the development of cardiovascular diseases such as atherosclerosis, heart failure, thrombosis and hypertension (52). It is intriguing that the interaction between diet and gut microbiota has garnered increasing attention. Components such as dietary polyphenols, dietary fibers, prebiotics, and probiotics found have been shown to enhance the growth of beneficial bacteria, improve lipid metabolism, and mitigate lipidemia, endotoxemia, inflammation, and endothelial dysfunction. Therefore, adhering to a long-term healthy dietary pattern has a beneficial effect on maintaining gut microbiota balance and preventing cardiovascular diseases (53). Indeed, certain medications used in the treatment of cardiovascular diseases also promote the formation of beneficial gut microbiota. Examples include statins, aspirin, and amlodipine (54). In addition to indirectly modulating the host gut microbiota using medications, research has explored fecal microbiota transplantation in mice to directly intervene in gut microbiota. The findings indicate that this intervention not only alleviates symptoms of diabetes but also reduces blood pressure and improves myocardial injury (55). Furthermore, indole-3-aldehyde has been demonstrated to alleviate atherosclerosis by reducing inflammation levels in endothelial cells (56). Similarly, indole-3-propionic acid has been found to mitigate the progression of atherosclerosis by promoting cholesterol efflux in macrophages (57). These studies indicate that, in addition to intervening in the kynurenine pathway, modulating tryptophan metabolism in the gut microbiota is also a highly promising approach for treating cardiovascular diseases.

## 3 The kynurenine pathway in cardiovascular disease

### 3.1 The kynurenine pathway and inflammation

Inflammation, recognized as a critical risk factor for cardiovascular diseases, progressively accumulates with advancing age. This chronic low-grade inflammation contributes to cellular aging and immune dysfunction, impairing the ability to mount appropriate immune responses to immune stimuli and exacerbating the burden of cardiovascular diseases (58). KYN and its downstream metabolites exert diverse effects on inflammation across different cell types. For example, kynurenic acid can diminish the generation of pro-inflammatory cells. It is noteworthy that the cytokine IFN-*γ*, recognized as the most potent inducer of KP activation in the human body, can induce the expression of IDO1 and kynureninase internally, thereby increasing the risk of developing arterial diseases (59). Research has shown that NAD^+^ in macrophages primarily originates from KP metabolism. Inhibiting IDO1 reduces NAD^+^ synthesis levels, simultaneously suppressing mitochondrial respiration and thereby inhibiting macrophage phagocytic function. Similarly, downstream of KP, quinolinic acid phosphoribosyl transferase can also modulate mitochondrial respiration and cellular immune function by influencing intracellular NAD^+^ levels in macrophages. With aging, declines in quinolinic acid phosphoribosyl transferase and NAD^+^ synthesis levels result in immune dysfunction, leading macrophages to adopt a phenotype characterized by weakened phagocytic capabilities and a pro-inflammatory state. Surprisingly, supplementation with quinolinic acid phosphoribosyl transferase and NAD^+^ enhances intracellular glycolysis and oxidative phosphorylation, restoring macrophages to a polarized state with robust phagocytic abilities and anti-inflammatory effects (60). Furthermore, the formation of foam cells in vascular inflammation has been found to be associated with AhR-induced upregulation of IL-8, IL-1β, and tumor necrosis factor-α (61).

Recent studies have demonstrated that the upregulation of IDO1 and increased KYN levels can lower blood pressure in mice under inflammatory conditions. Within endothelial cells, on one hand, IDO1, induced by the inflammatory cytokine IFN-*γ*, metabolizes tryptophan into KYN, which in turn activates the soluble guanylate cyclase (sGC) in its heme-free form, thereby facilitating coronary artery dilation through the cGMP-PKG pathway. On the other hand, kynurenine can induce vasodilation by enhancing cAMP levels in arteries through the activation of adenylate cyclase (62). Given that NO-mediated vasodilation relies on heme-containing sGC, yet under inflammatory conditions, sGC transforms into an enzyme lacking heme, rendering it insensitive to NO stimulation. This implies that IDO1 and KYN could serve as potential therapeutic targets for hypertension in systemic inflammatory diseases. Furthermore, IDO1 serves as a critical regulatory factor in atherosclerotic plaques and inflammation. Modulating IDO1 can effectively delay the progression of atherosclerosis and reduce systemic inflammation. Similar effects are also observed in pulmonary inflammation and inflammatory bowel disease (63).

In pathological conditions, IDO1 experiences strong induction by inflammatory mediators, resulting in a substantial increase in downstream metabolites of the kynurenine pathway. These downstream metabolites, including KYN, 3-hydroxykynurenine, 3-HAA, and QA, have demonstrated the capacity to induce apoptosis in various immune cells like T cells, B cells, NK cells, and neutrophils. Consequently, they aid in tempering inflammatory responses (64). Moreover, in atherosclerotic aneurysms, kynureninase and KMO exhibited significant elevation within macrophages. kynureninase demonstrated the ability to inhibit IL-6 expression and IDO1 through negative feedback regulation within the KP, consequently mitigating the inflammatory response (65). KYN orchestrates the immunosuppressive effect of AhR, countering the pro-inflammatory response during the acute inflammatory phase. However, it also contributes to promoting vascular disease through chronic inflammation (34). Presently, the KP operates as a double-edged sword in chronic inflammatory diseases, encompassing both pathogenic and compensatory mechanisms (66). Consequently, the role of KP in atherosclerosis remains a subject of debate. We postulate that the mechanism of KP in atherosclerosis may exhibit variability contingent upon distinct cell types. KP may exert diverse effects on disease progression concerning immunity and inflammation. These observations suggest the need for further exploration into the mechanisms underlying KP's involvement in atherosclerosis.

### 3.2 The kynurenine pathway and coronary atherosclerotic disease

Coronary artery disease (CAD) is a highly prevalent and fatal condition globally. It has been identified to have both familial and individual genetic predispositions (67). From a pathophysiological perspective, CAD is considered an inflammatory disease. Arteries, when exposed to diverse risk factors, accumulate substantial lipid deposits over time, forming plaques that gradually narrow the arterial lumen or cause arterial remodeling, possibly leading to rupture and, in severe cases, death (68). Multiple studies have indicated an association between elevated KYN concentrations and the risk of coronary artery disease (69, 70). There is clear evidence indicating increased levels of KYN, QA, and kynurenic acid in the plasma of patients with atherosclerosis (71). Furthermore, mice with atherosclerosis exhibit markedly elevated levels of IDO1 compared to wild-type mice, and serum IDO1 levels are positively correlated with the advanced stages of atherosclerosis (72). The pathogenesis of this disease may stem from T cell activation and IFN-*γ* release, leading to increased IDO1 levels. This process promotes macrophage apoptosis and foam cell formation, thereby advancing the progression of atherosclerosis (73). Another study found that activation of AhR in macrophages triggers the production of reactive oxygen species (ROS) via NADPH oxidase, leading to the generation of oxidized Ox-LDL, which is subsequently absorbed by macrophages and converted into foam cells (74). Therefore, we propose that during inflammation-induced stimulation, the release of IFN-*γ* upregulates IDO1 expression, accelerating tryptophan metabolism into kynurenine. Subsequently, this process further activates AhR in macrophages to produce ROS, exacerbating vascular inflammation, promoting lipid accumulation within blood vessels, and ultimately leading to atherosclerosis within the vasculature.

Additionally, research has shown that IFN-*γ* mediates the activation of AhR by KYN, leading to the suppression of transcription of glycolytic enzymes in vascular endothelial cells, thereby impairing glucose metabolism. Continuous depletion of NAD^+^ further drives cellular metabolic pathways towards fatty acid oxidation. Ultimately, metabolic dysregulation is accompanied by a pro-inflammatory shift in endothelial cell phenotype (75).

In the pathology of ischemic heart disease (IHD), cellular components such as T cells, macrophages, and mast cells play pivotal roles in atherosclerosis, potentially culminating in acute myocardial infarction (76). Recent Mendelian randomization studies have indicated a positive correlation between KYN and IHD (77). Moreover, IDO1 demonstrates a positive correlation not only with IHD but also with conditions like stroke, diabetes, and prostate cancer (78). The KTR emerges as a robust predictor of coronary events and mortality, as indicated by a large study involving 4,122 patients undergoing coronary angiography (79). Another cohort study involving 3,224 patients with stable angina unveiled that elevated urinary KTR correlated with an increased risk of major coronary events, acute myocardial infarction, ischemic stroke, and other adverse events in these patients (80). Although the observed effects of IDO1 in IHD contrast with the prior description, it remains plausible that these discrepancies arise as consequences of an acute cardiovascular attack, potentially representing a late-stage outcome of the disease.

Interestingly, in dendritic cells, IDO1 displayed a mitigating effect on atherosclerosis by fostering the proliferation and differentiation of Tregs *in vitro* through the KYN-AhR. It achieved this by elevating IL-10 expression, exerting an immunosuppressive influence (72). The competitive inhibitor of IDO1, 1-Methyltryptophan (1-MT), accelerates vascular inflammatory responses by inhibiting systemic IDO1. This inhibition triggers pivotal factors like vascular cell adhesion molecule-1 and monocyte chemoattractant protein-1, mirroring the increased accumulation of CD68 macrophages in atherosclerotic plaques and subsequently hastening atherosclerotic lesions. However, this heightened state can be reversed by administering 3-HAA, a downstream metabolite of KYN (81). These findings imply that IDO1 might exert a protective function in atherosclerosis by modulating the inflammatory response. They underscore the intricate and interconnected relationship between IDO1-mediated tryptophan metabolism and subsequent pathophysiological events in the context of atherosclerosis.In addition to its protective role, IDO1 also plays a promotive role in the development of atherosclerosis. Under the influence of Ox-LDL, macrophages induce IDO1 expression via the PI3K/Akt/NF-κB pathway, thereby promoting the production of inflammatory factors and foam cells. The application of the IDO1 inhibitor 1-MT can suppress these effects. This differential effect may be attributed to the upregulation of IDO1 in the early stages of atherosclerosis as a self-protective mechanism, while its function diminishes in the later stages leading to downregulation of IDO1 (49). Therefore, the precise regulation of IDO1 levels throughout the stages of atherosclerosis holds significant clinical significance in cardiovascular disease.

### 3.3 The kynurenine pathway and arterial calcification

Arterial calcification constitutes a systemic vascular disease prominently involving vascular smooth muscle cells (VSMCs) (82). It is primarily characterized by the accumulation of calcium phosphate salts within the arterial wall, exhibiting a high correlation with atherosclerotic plaque burden. Both IDO1 and IDO2 have been identified in bone marrow mesenchymal stromal cells (BMSCs). TRP has the capacity to stimulate BMSCs proliferation, yet elevated TRP levels may lead to excessive BMSCs proliferation. Conversely, heightened levels of KYN have been associated with bone loss by inhibiting BMSC activity, potentially contributing to osteoporosis (83). Moreover, Animal studies have shown that intraperitoneal injection of KYN inhibits osteoblast differentiation and enhances osteoclast resorption, leading to decreased bone formation and increased bone loss (83). Elevated peripheral levels of KYN activate the AhR-dependent pathway in both animal and human skeletal systems, leading to accelerated skeletal aging and bone loss in mice (84). As age progresses, the levels of IDO1 and KYN gradually elevate, potentially impacting osteoblast function through their influence on mitochondrial respiration (85). In a mouse model of atherosclerosis, the substantial reduction in the expression of osteogenic regulatory factors not only inhibits osteoblast differentiation but also mitigates arterial calcification (86).

In a study involving 325 patients diagnosed with moderate-to-severe chronic kidney disease, the downstream products of the KP, including QA, anthranilic acid, and HAA, exhibited positive associations with coronary artery calcification. Conversely, reduced TRP levels demonstrated a negative correlation with aortic calcification (87). Additionally, a decrease in the serum KTR has been observed in chronic kidney disease patients undergoing expanded hemodialysis treatment, subsequently resulting in decreased VSMCs calcification (88). Research by Liu Ouyang et al. revealed that The deficiency of IDO1 in VSMCs promotes the expression of the osteogenic transcription factor 2 (RUNX2), leading to enhanced osteogenic reprogramming of VSMCs. Animal experiments similarly demonstrate that intraperitoneal injection of KYN can inhibit the expression of RUNX2, thereby slowing down the progression of arterial calcification. This process is regulated by KYN, which modulates the interaction between the AhR and the E3 ubiquitin ligase component of cullin 4B, governing the proteasomal degradation of RUNX2 (89). These findings suggest that various substances within the KP may play distinct roles in the process of arterial calcification.

### 3.4 The kynurenine pathway and myocardial disease

Cardiac remodeling is a maladaptive response of the myocardium, characterized by changes in myocardial cell growth under the influence of mechanical stress and neurohormones. Without timely intervention, it can lead to irreversible myocardial hypertrophy and interstitial fibrosis, eventually resulting in severe impairment of cardiac function and death as heart failure symptoms worsen (90). Multiple pathogenic factors contributing to cardiac remodeling have been confirmed, with metabolomics data indicating a correlation between elevated plasma kynurenine levels and cardiac remodeling. Recent studies have shown that increased plasma kynurenine activates AhR, upregulating downstream genes associated with pathological left ventricular hypertrophy and fibrosis induced by pressure overload in cardiac myocytes and fibroblasts. However, supplementation with specific probiotics targeting the gut microbiota can modulate downstream target genes of the KYN-AhR axis, thereby reducing plasma kynurenine levels and alleviating ventricular remodeling (91). Yinhui Wang and colleagues investigated the involvement of IDO1 and KYN in pathologically hypertrophied myocardium. They observed elevated expression levels of IDO1 and KYN in the hypertrophied myocardium group compared to the control group. Additionally, these levels were notably higher in a group of mice with surgically induced myocardial hypertrophy, accompanied by an increase in downstream metabolites of the kynurenine pathway and a decrease in TRP levels (92). Through a series of *in vivo* and *in vitro* experiments, they demonstrated that knockout of IDO1 and down-regulating KYN could mitigate pathological myocardial hypertrophy by modulating the expression of GATA4 (GATA binding protein 4) via AhR regulation. Interestingly, subcutaneous injection of KYN significantly exacerbated myocardial cell hypertrophy in mice. Conversely, the application of an IDO1 inhibitor improved myocardial hypertrophy and remodeling. These findings further corroborate the hypothesis that myocardial hypertrophy is triggered by the activation of IDO1, which subsequently influences AhR-mediated myocardial hypertrophy, and also provide a theoretical basis for the development of IDO1- and KYN-related intervention drugs (92). Surprisingly, a rapid increase in the levels of IDO1 and KYN was observed in the hearts of neonatal mice following the removal of a portion of the heart. In neonatal cardiomyocytes, IDO1 was found to activate the SRC-YAP/ERK pathway through KYN binding to AhR, thereby promoting cardiomyocyte proliferation and regeneration (93). Furthermore, KYN demonstrated the ability to enhance the proliferation of vascular endothelial cells by inducing nuclear translocation of AhR in endothelial cells. This activation subsequently upregulated vascular endothelial growth factor, fostering cardiac regeneration (93).

Melhem et al. demonstrated a significant upregulation of IDO1 in a mouse infarction model. In mice with endothelial cell-specific deletion of IDO1, improvements in cardiac function and attenuation of ventricular remodeling were observed. Conversely, KYN induced the production of ROS by upregulating AhR, thereby promoting apoptosis in cardiac myocytes (94). Notably, there is limited research literature concerning the KP in myocardial diseases. Consequently, further exploration is warranted to elucidate the mechanism linking the IDO-KYN-AhR pathway with myocardial diseases. Effective treatment strategies for myocardial diseases by intervening in this pathway have emerged as a focal point in current research efforts.

## 4 Mechanisms of pathogenesis of the kynurenine pathway

In coronary atherosclerotic disease, the body upregulates IDO1 under the influence of the inflammatory cytokine IFN-γ, leading to the conversion of TRP into KYN, subsequently activating the downstream AhR. AhR triggers the production of ROS and Ox-LDL via NADPH oxidase. Subsequently, macrophages phagocytize Ox-LDL, transforming into foam cells and contributing to the progression of atherosclerotic disease. Similarly, various tumor cells within the human body have demonstrated that KYN can activate AhR in an autocrine or paracrine manner, further driving cancer progression (95). Moreover, kynurenic acid exacerbates vascular inflammation and accelerates atherosclerosis development by reducing IL-10 levels through activation of the cAMP-dependent pathway and inhibition of Erk1/2 phosphorylation (96). Furthermore, the application of the IDO1 inhibitor 1-MT has been shown to increase vascular inflammation, exacerbating atherosclerosis. Interestingly, downstream metabolite of KYN, 3-HAA, has been found to downregulate vascular cell adhesion molecule-1 and monocyte chemoattractant protein-1, while simultaneously reducing the infiltration of CD68 macrophages within arterial plaques, exerting a protective effect against inflammatory responses (97). Differentiating from other types of cells, it is only observed in endothelial cells that the absence of IDO1 can reduce cardiomyocyte apoptosis. Additionally, mice with endothelial cell-specific deficiency in IDO1 exhibit a significant improvement in cardiac function (94). It is noteworthy that in a mouse model of nephrotoxic nephritis, IDO1 is significantly elevated, and the administration of 1-MT leads to the accumulation of CD4T cells and macrophages in the kidney, exacerbating crescentic glomerulonephritis (98). Additionally, indole metabolites derived from tryptophan metabolism have been identified to induce leukocyte activation via AhR, aggravating inflammation and increasing the risk of thrombosis (99).

**Figure 2:**
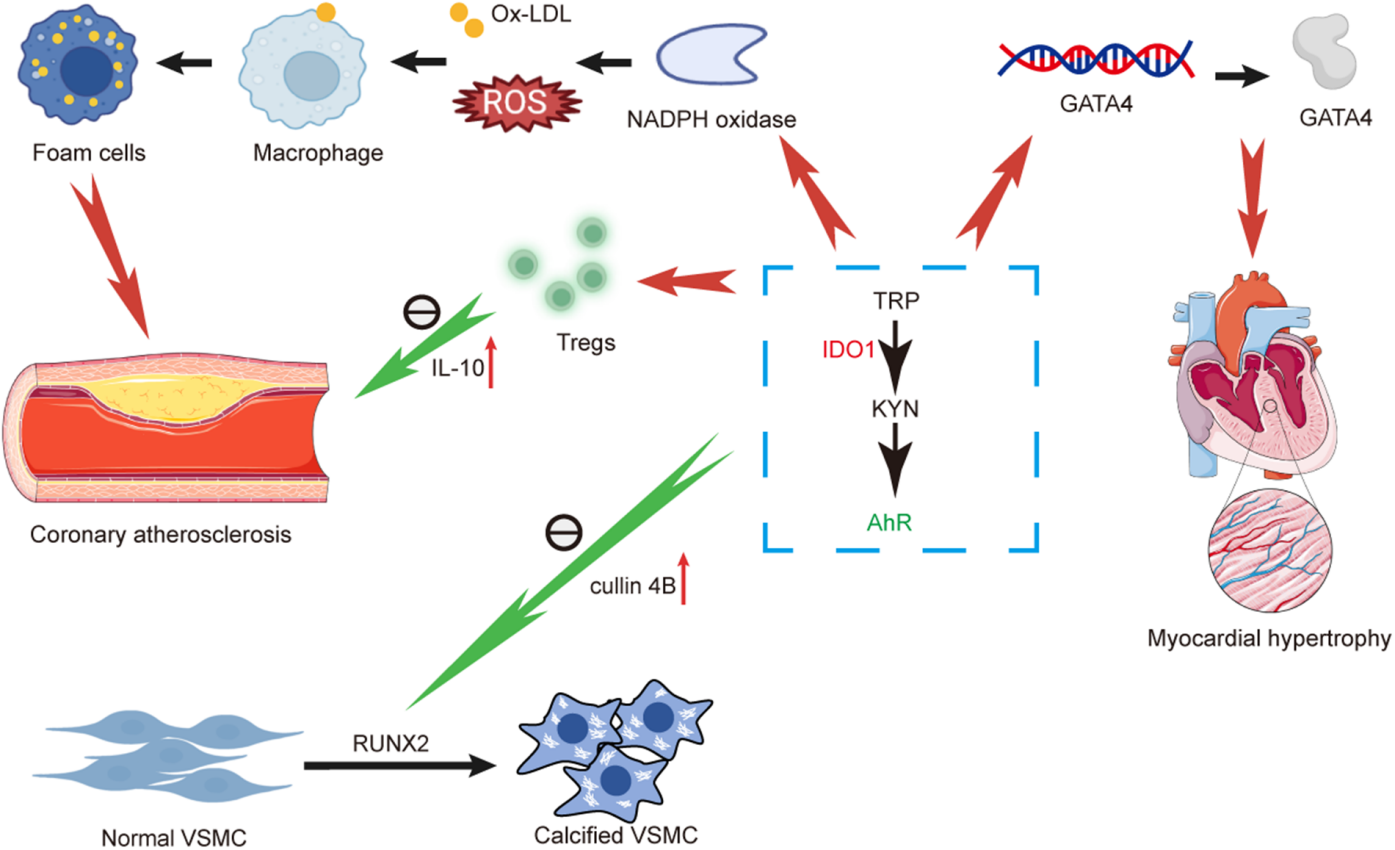
Diagram illustrating and summarizing the pathophysiological mechanisms proposed for mechanisms linking kynurenine pathway and coronary atherosclerosis, arterial calcification and myocardial hypertrophy. AhR, aromatic hydrocarbon receptor; GATA4, GATA binding protein 4; TRP, tryptophan; IDO1, indoleamine-2,3-dioxygenase-1; IL-10, interleukin-10; KYN, kynurenine; NADPH, nicotinamide adenine dinucleotide phosphate hydrogen; ROS, reactive oxygen species; Ox-LDL, oxidized low density lipoprotein; VSMC, vascular smooth muscle cell.

In VSMCs, the loss of IDO1 and deficiency in kynurenine induce upregulation of RUNX2 by altering the binding between AhR and cullin 4B, ultimately prompting VSMCs osteogenic reprogramming and arterial calcification. Finally, in myocardial diseases, the IDO1-KYN-AhR pathway exacerbates pathological myocardial hypertrophy by modulating GATA4. Conversely, in younger hearts, KYN-AhR upregulates genes associated with cardiac hypertrophy and fibrosis, influencing ventricular remodeling under stress and leading to cardiac hypertrophy and fibrosis. Intriguingly, IDO1-KYN-AhR not only indirectly promotes cardiomyocytes via YAP/ERK but also directly stimulates vascular endothelial cell proliferation, contributing to cardiac regeneration (Figure 2).

## 5 The kynurenine pathway in cardiovascular diseases diagnosis and prognosis

Research evidence suggests that downstream metabolites of the KP serve as risk factors for various cardiovascular diseases, including atherosclerosis, hypertension, and vascular inflammation. In a cohort study on CAD, it was found that, besides certain inflammatory markers, KYN, KTR, anthranilic acid, and 3-hydroxykynurenine were positively associated with coronary artery death events, while TRP and xanthurenic acid were negatively correlated (71). Elevated levels of KYN and KTR serve as predictive factors for coronary heart disease and are associated with disease severity. Increased KYN and KTR levels along with decreased TRP levels are also associated with the occurrence of major adverse cardiac events (100). Moreover, metabolomic analysis of serum samples from patients with heart failure, with or without reduced muscle endurance, revealed that KYN can serve as an independent predictor of heart failure combined with reduced muscle endurance. This suggests a significant correlation between skeletal muscle energy metabolism and serum metabolites in patients with heart failure (101). In stable angina populations, elevated plasma levels of KYN indicate a significantly increased risk of subsequent acute myocardial infarction (79). Additionally, a longitudinal study spanning 7 years demonstrated that urinary KTR levels can serve as predictors of type 2 diabetes mellitus in patients with CAD (9). This association may be explained by the correlation between coronary artery disease and insulin resistance (102). In a plasma metabolomics study of patients with right heart-pulmonary vascular dysfunction, strong correlations were observed between KYN, kynurenic acid, anthranilic acid, QA in the KP and pulmonary arterial hypertension. Moreover, experiments conducted in mice confirmed an increase in IDO1 levels in the context of pulmonary arterial hypertension, suggesting that specific enzymes and metabolites within the kynurenine pathway may serve as promising biomarkers for discerning abnormal pulmonary vascular function (103).

In addition to metabolites within the KP, downstream indole derivatives of TRP in plasma, such as indole-3-propionic acid and indole-3-aldehyde, have also been found to be negatively correlated with advanced stages of atherosclerosis. Moreover, they serve as effective predictors of adverse cardiovascular events following surgery in patients with atherosclerosis (104). Current research suggests that KYN may serve as a novel biomarker and therapeutic target in cardiovascular diseases, with elevated plasma levels of KYN indicating an increased risk of adverse cardiovascular events. Certain downstream metabolites of the KP may also serve as monitoring indicators for conditions such as heart failure and acute myocardial infarction. However, there is still a lack of robust evidence regarding the detection standards and normal concentration ranges for KP downstream metabolites, necessitating further validation through large-scale studies.

## 6 The therapeutic applications of the kynurenine pathway in cardiovascular diseases

Compared to the general population, IDO1 activity is elevated in individuals with obesity, with a more pronounced effect observed in premenopausal women, further amplifying the interaction between them and exacerbating the risk of cardiovascular diseases (105). Additionally, the role of IDO1 in atherosclerosis is currently uncertain, with some evidence suggesting a protective function of IDO1 in modulating atherosclerosis and inflammation. Induction of IDO1 expression may alleviate inflammatory responses by inhibiting T-cell proliferation and apoptosis (81, 106). Interestingly, another study suggests that inhibiting IDO1 can improve insulin resistance, alleviate inflammation, and enhance lipid metabolism in the liver and adipose tissue by modulating the gut microbiota. Surprisingly, these effects are not attributed to a reduction in KYN and its downstream metabolites following IDO1 inhibition. Instead, TRP undergoes metabolic conversion into indole derivatives, which further activate the AhR. This activation helps maintain gut immune homeostasis while simultaneously reducing systemic inflammation and the heightened risk of cardiovascular metabolic disorders associated with elevated KYN levels (107). Therefore, the underlying mechanisms of this difference may be linked to the regulation of the gut microbiota. Considering that changes in IDO1 may also be influenced by the stages of atherosclerotic disease and that IDO1 and the disease may be mutually causative, investigating IDO1-related drug mechanisms at various stages of atherosclerosis is imperative. Despite lingering uncertainties, multiple studies have demonstrated that the application of IDO1 inhibitors can delay the progression of atherosclerosis, affirming that inhibiting IDO1 can indeed serve as a novel therapeutic target for cardiovascular diseases (108). Additionally, during the early stages of cardiac arrest, activation of the KP in plasma indicates adverse neurological outcomes. The application of IDO1 inhibitors can mitigate brain damage after cardiac arrest and improve survival rates following cardiac resuscitation (109). Although research on IDO1 inhibitors has progressed to phase III clinical trials, the controversy surrounding their mechanisms in cardiovascular disease has prevented clinical trials using IDO1 inhibitors for treating cardiovascular diseases. Therefore, a substantial amount of precise basic research is still needed to guide subsequent clinical translation of these drugs. It is worth considering that studying enzymes or metabolites downstream of IDO1 and developing corresponding drugs may provide a more targeted treatment approach (110).

In addition to regulating inflammation, induction of IDO1 expression in pulmonary endothelial cells has been shown to reverse pathological vascular proliferation and modulate VSMC phenotype transformation, thereby attenuating vascular remodeling in pulmonary arterial hypertension (111). Research in the field of cardiac transplantation has shown that intramyocardial injection of adenovirus carrying IDO1 significantly prolongs the survival time of allogeneic cardiac transplants in mice. Moreover, the transcription levels of inflammatory factors are significantly downregulated. Interestingly, the effect is further enhanced when IDO1 is combined with 3-HAA (112). Additionally, modulating IDO1 levels in human cardiac stem cells may prolong the survival time after transplantation by inhibiting T lymphocyte proliferation, thus opening up new avenues for treating myocardial infarction.

In addition to IDO1, 3-HAA, a downstream metabolite of the KP, has been identified as a novel drug with anti-inflammatory properties and the ability to regulate lipid metabolism, leading to decreased plasma cholesterol and triglyceride levels. Although existing research suggests that the beneficial effects of 3-HAA on the body occur under conditions beyond physiological levels, it underscores the potential therapeutic value of alleviating inflammation and atherosclerosis by modulating 3-HAA or upstream kynureninase (48). As a downstream metabolite of KYN, anthranilic acid has been found to increase the production of IL-10 in B cells within the spleen when its synthetic derivative, 3,4,-Dimethoxycinnamoyl anthranilic acid, is orally administered. Additionally, this compound alleviates arterial inflammation and reduces the production of cytokines. Importantly, experiments using KYN alone do not produce these effects, suggesting that the protective role in atherosclerosis is attributable to downstream metabolites of KYN rather than KYN itself (113). Furthermore, studies have demonstrated that inhibiting KMO in mice can significantly reduce mortality rates following acute viral myocarditis by alleviating the production of various inflammatory chemokines (114). Despite the close association between many metabolites of the KP and cardiovascular diseases, there is currently a lack of systematic foundational research linking them together comprehensively for potential use in subsequent clinical treatments.

## 7 Summary and prospects

KP, a crucial part of tryptophan metabolism, has garnered significant attention in cardiovascular disease research. This paper serves to outline the pathway's physiological functions, its downstream metabolites, and their intricate associations with various cardiovascular conditions. These metabolites not only act as potential biomarkers but also wield influence over cardiovascular health through diverse mechanisms.In coronary artery disease, the KTR emerges as a pivotal predictor of coronary events and mortality. While IDO1 exhibits a protective role against atherosclerosis in its early stages, it also promotes the progression of atherosclerosis in later stages. Within arterial calcification, KP metabolites tightly correlate with the activation of vascular smooth muscle cells and osteoblast differentiation. Additionally, the kynurenine pathway significantly impacts cardiomyopathies, influencing critical aspects like cardiomyocyte proliferation, hypertrophy, and fibrosis.

Despite notable research strides, the specific mechanisms of KP in some cardiovascular diseases remain contentious and warrant further exploration. Future studies should delve into the pathway's intricate roles across diverse cardiovascular conditions to uncover potential therapeutic targets. Exploring the interplay between KP and other metabolic pathways offers promising prospects for multi-treatment approaches.Comprehensive research in this domain is poised to deliver breakthroughs in diagnosing and treating cardiovascular diseases, promising improved health and enhanced quality of life for patients in the future.
